# Machine learning based skin lesion segmentation method with novel borders and hair removal techniques

**DOI:** 10.1371/journal.pone.0275781

**Published:** 2022-11-10

**Authors:** Mohibur Rehman, Mushtaq Ali, Marwa Obayya, Junaid Asghar, Lal Hussain, Mohamed K. Nour, Noha Negm, Anwer Mustafa Hilal

**Affiliations:** 1 Department of Computer Science & Information Technology, Hazara University, Mansehra, Pakistan; 2 Department of Biomedical Engineering, College of Engineering, Princess Nourah bint Abdulrahman University, Riyadh, Saudi Arabia; 3 Faculty of Pharmacy, Gomal University, D I Khan, Pakistan; 4 Department of Computer Science and Information Technology, King Abdullah Campus Chatter Kalas, University of Azad Jammu and Kashmir, Muzaffarabad, Azad Kashmir, Pakistan; 5 Department of Computer Science and Information Technology, Neelum Campus, University of Azad Jammu and Kashmir, Athmuqam, Azad Kashmir, Pakistan; 6 Department of Computer Sciences, College of Computing and Information System, Umm Al-Qura University, Mecca, Saudi Arabia; 7 Department of Computer Science, College of Science & Art at Mahayil, King Khalid University, Abha, Saudi Arabia; 8 Department of Computer and Self Development, Preparatory Year Deanship, Prince Sattam bin Abdulaziz University, Al-Kharj, Saudi Arabia; National Institute of Technology Uttarakhand, INDIA

## Abstract

The effective segmentation of lesion(s) from dermoscopic skin images assists the Computer-Aided Diagnosis (CAD) systems in improving the diagnosing rate of skin cancer. The results of the existing skin lesion segmentation techniques are not up to the mark for dermoscopic images with artifacts like varying size corner borders with color similar to lesion(s) and/or hairs having low contrast with surrounding background. To improve the results of the existing skin lesion segmentation techniques for such kinds of dermoscopic images, an effective skin lesion segmentation method is proposed in this research work. The proposed method searches for the presence of corner borders in the given dermoscopc image and removes them if found otherwise it starts searching for the presence of hairs on it and eliminate them if present. Next, it enhances the resultant image using state-of-the-art image enhancement method and segments lesion from it using machine learning technique namely, GrabCut method. The proposed method was tested on PH2 and ISIC 2018 datasets containing 200 images each and its accuracy was measured with two evaluation metrics, i.e., Jaccard index, and Dice index. The evaluation results show that our proposed skin lesion segmentation method obtained Jaccard Index of 0.77, 0.80 and Dice index of 0.87, 0.82 values on PH2, and ISIC2018 datasets, respectively, which are better than state-of-the-art skin lesion segmentation techniques.

## 1. Introduction

The World Health Organization (WHO) reported that one out of three cancers, is diagnosed as skin cancer [1]. According to a survey [2], one person passes away after each 4 minutes from skin cancers globally. The skin cancer diagnosing rate reported per day in US is approximately 9500, and its mortality rate per hour is 2 [3]. The most familiar type of skin cancer is known as melanoma that grows from the pigment-producing cells known as melanocytes. The melanoma skin cancer cases increased by 47% globally since 2010. In Europe, and Australia the number of annually diagnosed melanoma cases are more than 100,000, and 15229, respectively. The non-invasive method termed as dermoscopy is commonly used to examine the skin surface [4] for diagnosing melanoma. However, examining the dermoscopic images manually is inefficient and its diagnosing rate is unsatisfactory [5–7].

To improve the speed and accuracy of the melanoma diagnosis, the CAD systems are introduced [4]. The CAD systems segment the lesion(s) from the skin image and diagnose melanoma skin cancer in the segmented lesion(s), However, the melanoma diagnosing accuracy of the CAD systems is not up-to the mark due to nonavailability of most effective skin lesion segmentation algorithms dealing with skin images containing artifact like hairs, and corner borders [4, 5].

**Fig 1:**
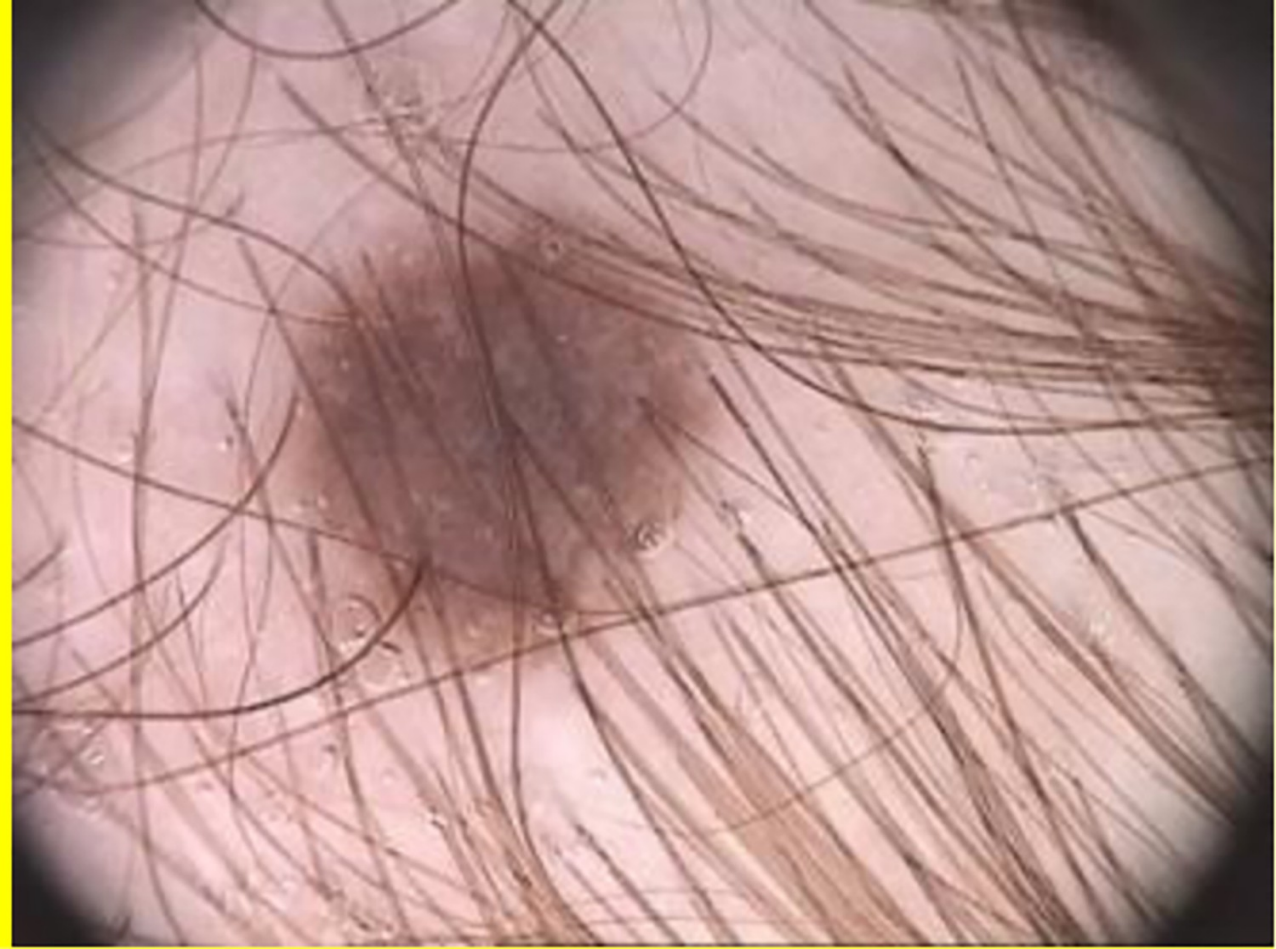
Dermoscopic image containing corner borders and hairs.

A number of skin lesion segmentation methods like [4, 5, 8–24] have been proposed for improving the segmentation results of skin lesion. Among these skin lesion segmentation methods, the methods [5, 8, 11, 18] work fine for dermoscopic images with no hair, and corner borders (i.e., pre-processed dermoscopic images). However, they often generate false results in case of the dermoscopic image containing hairs and/or corner borders and lesion(s) as one shown in Fig 1. The reason behind is the fact that the corner border(s) are most often considered as lesion(s). Additionally, these methods have no potential to remove hairs if any on the dermoscopic image.

The methods [10, 13, 19, 23] remove hairs from the dermoscopic images first and then segment the lesion from the dermoscopic. However, they blur the dermoscopic image after hair removal which in turn degrade the segmentation accuracy. Furthermore, they are unable to remove corner borders prior to lesion segmentation. As a result, their lesion segmentation accuracy is poor for dermoscopic images containing corner borders.

The method proposed in [4, 9, 14] have the potential to deal with dermoscopic images containing hairs and/or corner borders. The former two methods use binary mask, while the later one uses the “imclearborder” function of MATLAB for the removal of corner borders in the dermoscopic images. The results’ accuracy of these methods is not satisfactory for dermoscopic images containing corner borders with color similar to lesion(s), or varying size corner borders. Furthermore, their hair removal results’ accuracy is not satisfactory for images with low contrast between hair and its background. Additionally, they blur the image during the hair removal process which in turn affect the lesion segmentation results.

An effective skin lesion segmentation method is proposed in this research work to improve the skin lesion segmentation results in case of dermoscopic images containing hairs having low contrast with surrounding background and/or varying size corner borders with color similar to lesion(s,. The working of the proposed method is described as follows.

The proposed method removes corner borders and hairs from the given dermoscopic RGB skin image using novel corner borders and skin hairs removal techniques. The resultant image is then transformed into HSV color and the contrast of its V channel is improved by using image enhancement method [25]. Next, the two channels, i.e., H, S, and improved version of V channels are merged, and log exponential transformation is applied over the newly formed image to further improve its contrast. A GrabCut (foreground extraction technique) is then applied to extract lesion from the enhanced image.

### A. Contributions

The contributions of our research work are described as follows.

1. We identified two limitations of the available skin lesion segmentation methods: a) the accuracy of the available corner borders removal methods used in the context of skin lesion segmentation is not satisfactory for skin dermoscopic images containing varying size corner borders or corner borders with color similar to lesion,
  b) the available hairs removal methods blur the skin dermoscopic image after hair removal.
2. A novel corner borders removal method based on locating the extreme inner and extreme outer contour of the corner borders is proposed, and
3. A skin hair removal method based on mask inpainting is introduced.

### B. Paper organization

The rest of the paper is organized as follows: the related work is provided in Section 2, and the proposed lesion segmentation method details are described in Section 3. The description of dataset used for quantitative comparison of our method with six latest lesion segmentation algorithms is given in Section 4. The Section 5 provides paper’s conclusion.

## 2. Related work

This section provides detailed information regarding the available skin lesion segmentation techniques.

In [8], basic adaptive thresholding with skew estimation technique is used to find infected area in the 0 stage melanoma images. The basic idea in this method is to find an effective threshold in the narrow range use for segmenting the infected region in the skin image. To compute the adaptive threshold, it first takes the Upper Bound (UB) and Lower Bound (LB) of the threshold of the extracted infected region and computes the mean (M) of the extracted region. Next, it computes the average mean of all infected regions in the dataset. The average mean is then compared with the mean (M) of an image and if both are found similar then the previously defined limits of the threshold are used for segmentation. Otherwise, skewness information is used to find the amount of shift average mean forward to the mean (M) of the image. The skewness information finds the center weight of the histogram, with help of this numerical value the UB and LB are updated. These updated values are used to segment the image. However, its results’ accuracy is not up to the mark for images holding corner border, and/or hairs.

In [10], a lesion detection method is proposed. In this method, the hairs is removed by replacing the hairs pixels with non-hair pixels and smooth the final result by applying the median filter. Next the resultant RGB image is transformed into HSI, HSV, and LAB color spaces. All channels of each of the aforementioned color spaces are extracted. After this, other combinations of color channels are formed by applying logical OR operation over the two channels belonging to different color spaces. In this way, 25 different color channels are formed. One of these 25 channels is selected for the segmentation based on visual analysis. Next, the external artifacts like skin lines, air bubbles, or other random noise are removed from the selected channel. After this, smoothing with the help of a circular averaging low-pass filter is applied to the resultant image. Next, enhancement or intensity adjustment is performed in such a way, that the values of pixels in the image are mapped into a new range. The purpose of this mapping is to smooth and stretch the image histogram, to find a more suitable value in the thresholding step. The enhanced image is segmented with the help of otsu’s method [20] into two clusters. One of these clusters refers to the foreground, i.e., lesion, while the other denotes the background, i.e., surrounding area. An algorithm used for finding the threshold value with the help of discriminate analysis is applied. In this analysis, zeroth and first-order cumulative moments of the histogram are measured and used to define the separation level of these two clusters. Next, the optimal threshold value is calculated from the minimization of within-cluster variance. The within-cluster variance is the weighted sum of the variance of two clusters. The resultant binary image has a value of 1 for all those pixels which are greater than the threshold level and 0 for all other pixels. The connected components like the lesion and background, are then determined and labelled using run-length encoding technique. Subsequently, the two major areas, i.e., lesion and its surrounding remain, and other all components are discarded. Finally the lesion boundary holes are filled with the help of morphological filling operations. However, the use of median filter after hairs removal process most often blurs the image which makes the process of lesion segmentation complicated. Moreover, it has no potential to eliminate the borders.

Two algorithms: adaptive thresholding and K-Mean clustering are used in [9] for detection of skin cancer. The adaptive thresholding converts the input image into grayscale. The grayscale image is then enhanced using the image adjustment technique and converts into binary. Next, the binary image is inverted to create the ROI. The segmentation is then started from the center of the region. The process of segmentation is continued until the whole lesion boundary is covered. The aforementioned operations create a boundary matrix which is then used for filling inside when the tumor is identified. In the end, the segmentation result is compared with ground truth. The K- Mean clustering divides the image into two clusters (when the image has no borders) or three clusters (when the image has borders). The number of clusters is dependent on the mask calculated in the pre-processing step of [26]. Four different parameters i.e., hammoude distance, true detection rate, false position rate, and cross error are then used to evaluate the segmentation results. However, the border removal accuracy of this algorithm is not satisfactory for images containing varying size corner borders. Furthermore, its border removal method is computationally expensive because it employs spatial filtering over the whole image. Additionally, the hair removal process is shown in the flow diagram but its details are not provided.

In [11] the proposed method used the fusion threshold method for lesion border detection. This fusion threshold method joins four different threshold methods, i.e., Huang & Wang’s fuzzy similarity method [18], Kapur et al.’s maximum entropy method (LIANG-KAI HUANG et al. 1995), Kittler & Illingworth’s minimum error thresholding method [23], and Otsu’s clustering-based method [24], and the goal of this is to obtain best results of the segmentation that is independent of image statistical properties. The proposed method uses only the blue color channel from the RGB color space and applies each threshold method of the ensemble to the blue color channel image to generate the set of threshold images. After this, an energy function is used to control the unreliable decision at pixel level during the thresholding process. The four borders are then obtained by filling output of binary fusion and extracting the largest four connected components from it. However, the results‘ accuracy of this method is not satisfactory for images with hairs and/or oil bubbles.

The proposed method in [5] uses the GrabCut algorithm for skin lesion segmentation. This method first performs K-mean clustering on the input RGB image. The RGB is then transformed into HSV color. Next, the color channels of HSV color space are split and adaptive histogram equalization is performed on each color channel. The resultant channels are then merged. After this, the GrabCut algorithm starts the process of segmentation with a mask or rectangle depending on the threshold value, i.e.,65. In mask-based technique, a mask is generated with a threshold as the probable lesion area. However, if the generated mask surpasses the threshold value, then a rectangle is used to carry out the lesion segmentation process. However, pre-processed images are required for the proposed method. The pre-processed images means all the artifacts like image borders, and skin hair are removed from the image priory. The lesion segmentation results of this method are poor for images containing artifacts.

In [24], very deep residual network-based technique for the detection and classification of melanoma is proposed. This technique is composed of two phases. In the first phase, a Full Convolutional Residual Network (FCRN) is used to segment the skin lesion from the image. The FCRN based on residual blocks takes an image as input and produce equal-sized predication masks. After this successful down sampling operation, the feature map dimensions are reduced as compared to the original image size. To overcome this gap a deconvolutional layer is added which performs the up-sampling to get equal-sized predication maps with input image size. The network produces many skin lesion prediction maps by using different level features and then combines these maps with adding operation defined by the deconvolutional layer. As an outcome, the maps containing local and global features of the skin lesion are produced. In the second stage, classification is performed with the help of two classifiers, i.e., softmax and support vector machine (SVM) producing the two initial results. After this, the average of these results is computed and considered as a final result. In addition, CNN is used for lesion segmentation in [20–22, 27]. However, the results’ accuracy of CNN is not satisfactory because of problems: it needs a huge labelled dataset to generate accurate results, and its training takes too much time for large dataset. In addition, the method fails to segment when low contrast occurs between the lesion and normal skin.

In [4], wavelet transform and morphological operations are used to extract lesion area from the skin image. This method performs three steps to extract lesion from the skin image. The image enhancement and hair removal are carried out in pre-processing (first step). In image enhancement active contour is used to locate the infected area in the RGB image, and its blue channel is used for further processing. The otsu’s method-based thresholding is then applied over the blue channel to make the distribution of color equal in the image. The hair is then removed from the resultant image. The binary mask is then created by employing otsu’s thresholding method which is then used for removing the corner borders. The segmentation using Discrete Wavelet Transformation (DWT) is then carried out in the second step. In this step, the image is decomposed into horizontal, vertical, diagonal, and approximation bands. The approximation band is then further decomposed into the H, V, D, and A and the required result is obtained. In third step, i.e., post-processing, the average filter is applied to smooth the boundaries of the obtained segmented result. However, its borders removal is not adequate for images containing varying size corner borders. Moreover, the results of the hair removal method used are not satisfactory in images containing low contrast between the hair and its background. Additionally, it blurs the image after hair removal.

In [13], the Delaunay Triangulation method is used for the segmentation of melanoma images. In first step, morphological transformation operations are used to remove hairs from the input image. The histogram equalization is then employed on the Y channel of the input image to expose the border of lesion. Two parallel segmentation processes are then employed for detection of skin and lesion, respectively. In the first process, the input image is transformed into YCrCb color and the skin area is located using thresholding. The output image is then converted into HSV and thresholding is performed again to generate the new image containing only the lesion area. The second parallel process applies a Gaussian blur filter on each channel of the image and the resultant channels are then combined. The canny edge detection is then applied to create an edge image. The Delaunay Triangulation method takes edge image as input and finds its triangulation graph. The region association method is then used for the segmentation of triangulation graph. Transform. However, this method has no potential to remove borders located at the corners of the dermoscopic images.

In method [20], the skin lesions are segmented by applying perceptual color difference saliency algorithm. This method is composed our modules. The first module named as color image transformation transformed the RGB into CIE XYZ image which is then converted to CIE L*a*b* color space image. This mage is then passed to luminance image enhancement function (second module) that improve the luminance of the image without affecting the pixels’ original color. Next, the pixel saliency is determined by using mean of background color and object color in the salient pixel computation (third module). The standard deviation and background mean are determined from of an ellipsoidal patch’s pixels values designed by midpoint algorithm, while the object mean is determined from pixels of the rectangle patch. Finally, the extra elements that might be remaining after the segmentation are removed using median filter. The MATLAB built in function is used for image border removal. The lesion segmentation results of this method are poor in the case of images containing varying size corner borders.

In [15], the skin lesion was segmented by Convolutional Neural Network (CNN) based U-net algorithm. The performance of this algorithm was tested on ISBI 2016 dataset containing skin microscopic images without borders and hairs. In [16] CNN with auxiliary information was used for skin lesion segmentation. However, the ISBI 2017 dataset contain skin lesion images without borders.

In [17] GrabCut algorithm was used for segmenting the skin lesion from dermoscopic images. However, it was trained and tested on ISIC dataset containing images with no corner borders.

In [23], the hair is removed from the dermoscopic image first. The SLIC is then employed on the hair free image to generate similar clusters. The resultant grayscale clustered image is then provided to ACO to detect edges. The lesion area is then segmented using the concepts of convex hull and thresholding. The CNN is trained on the segmented lesion images to classify them as malignant or benign. This hybrid method works fine for images containing hairs, but its results is poor for images containing borders because it often detects borders as lesions.

In [19], the hairs and markers present on the dermoscopic image are removed using threshold and morphological processing at pre-processing level. The resultant pre-processed image is then segmented using K-mean clustering. The Firefly algorithm is employed over the segmented image for determining the threshold value for eliminating the false pixels present in the segmented image. the proposed method got an outstanding accuracy, i.e., 98.9% on the images of PH2. However, this method lacks the capability of borders removal, as a result its accuracy is unsatisfactory in case of dermoscopic images containing borders.

In contrast to the aforementioned lesion segmentation methods, our proposed method excludes corner borders using their extreme outer and inner contours and eliminates hairs using a mask inpainting method which comparatively generates less amount of blurriness in the resultant image. The resultant blur image is then enhanced by employing state-of-the-art enhancement method. Finally, the lesion region is segmented with the help of the GrabCut method.

## 3. Proposed method

**Fig 2:**
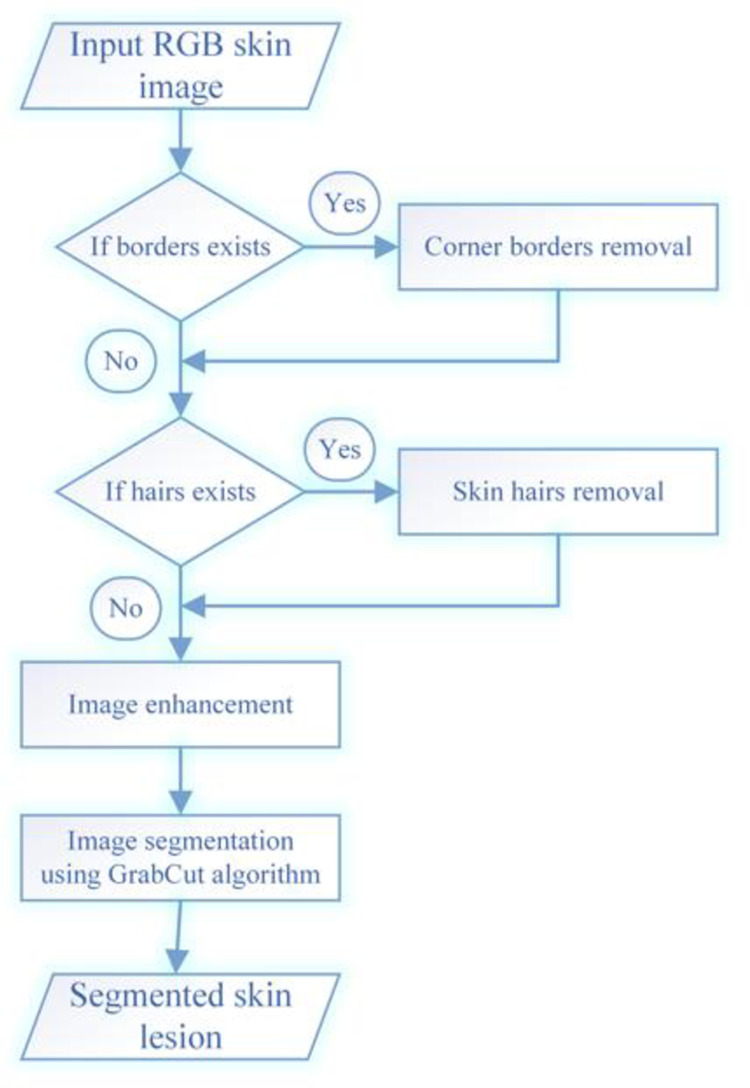
Flow diagram of our proposed skin lesion segmentation method.

This section present the working details of our proposed method. The proposed method takes RGB skin image as input and eliminates its corner borders if any. Next, the skin hairs over the image is searched and removed. The resultant image is then enhanced and GrabCut algorithm is employed for lesion segmentation. The flow diagram of the proposed method is shown in Fig 2.

### A. Corner borders removal

**Fig 3:**
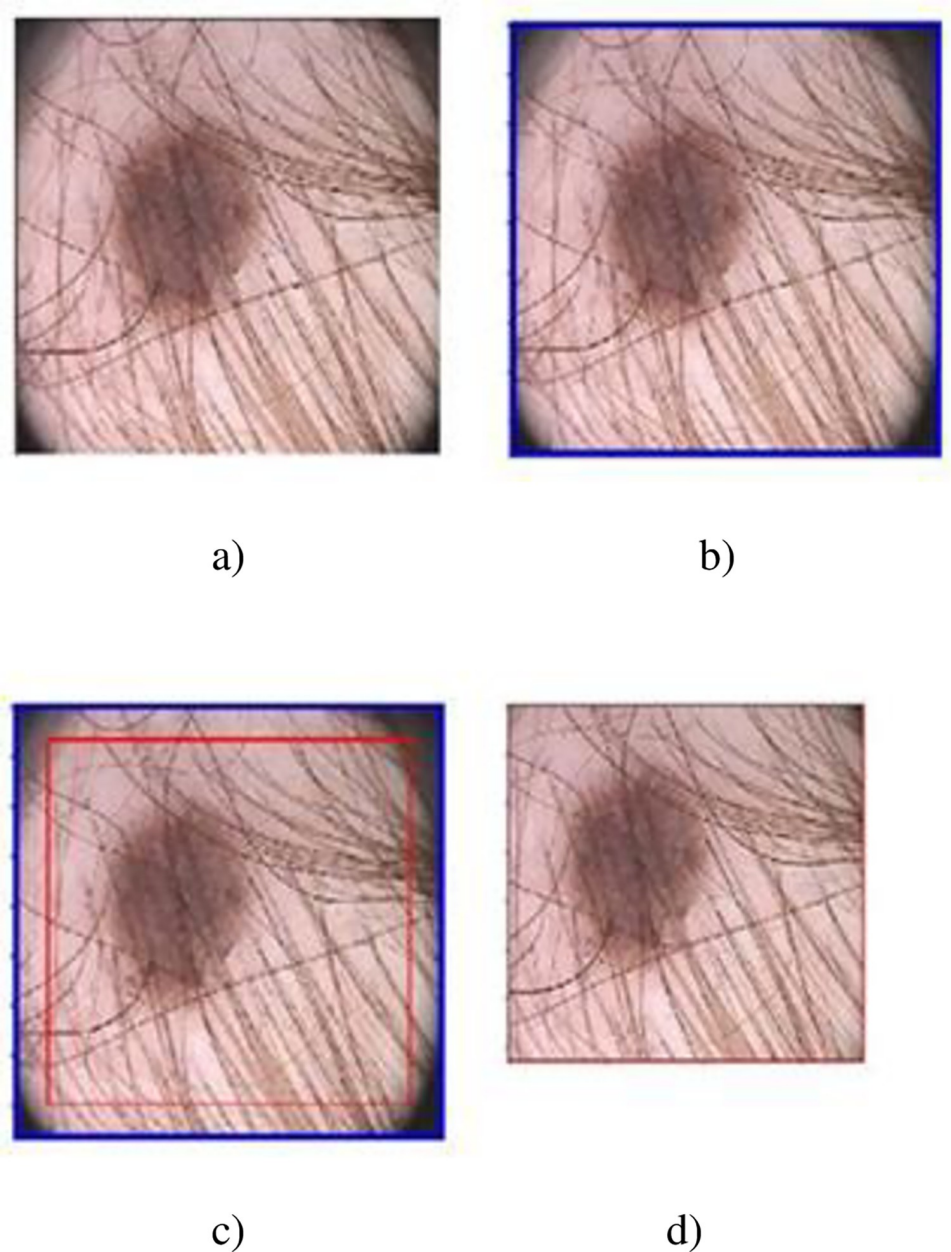
a). Input image with corner borders, b) extreme outer contour of corner borders, c) the extreme inner contour of corner borders, d) the result after corner borders removal.

The devices like dermatoscope, and microscope used by dermatologists for examining skin lesion have round shape lens which captures irrelevant information like border with images. Most of the dermoscopic images in PH2 dataset contain similar color lesion and corner borders which often cause false detection. The goal of this module is to eliminate the corner borders of the skin image to reduce false detection rate of lesion. To achieve this aim, the extreme outer and inner contours of the corner borders are determined and the area occurring between these rectangles are detached from the given RGB skin image. The extreme outer contour is computed by employing a simple contour approximation method. This method locates the position of the endpoint (pixel) of the corner borders overlaid on the input skin image. The computed positions are then placed in a vector. The extreme outer contour is then drawn with the help of these stored positions. For example, the endpoint positions of the corner borders of an image shown in Fig 3(A) are specified below.


[[0,0],[0,434],[434,434],[434,0]]

Fig 3(B) shows the extreme outer contour of the corner borders located with help of stored positions. To locate the extreme inner contour of the corner borders, a gray level threshold (T), and the initially estimated endpoint of the corner border are used. It is found in the training process that using gray level 4 as threshold is comparatively better in locating the corner borders’ ending pixel. Moreover, it is also noted that the corner borders’ length and width are less or equal to 20 pixels. Therefore, [20, 20] is taken as the initially estimated endpoint of the corner border. The initially estimated endpoints of the corner borders and the specified threshold are then utilized to locate the left top, left bottom, right top, and right bottom endpoints of the inner rectangle.

To compute the coordinate of left-top end point of the inner rectangle, the threshold value is compared with the estimated endpoint value and two of its previous pixels in the left-up word direction. If the threshold value is greater than these three pixels values then that point is assumed to be the inner rectangle left-top. In contrast, if the threshold value remains the same till the image starting pixel then that image is to be declared to have no top-left border. In such scenario, this module will not search for locating the other three border corners but rather it will finish the working of this module and move towards next module. In case if it found the coordinate of the left corner border then it will proceed towards finding the coordinates of other corner borders. This procedure is implemented using Eq (1)

$$b=\left\{ \begin{array}{l} (i,j)\;if\;(I(i,j)<T\\ \;and\;I(i-1,j-1)<T\\ \;and\;I(i-2,j-2)<T\\ i=i-1,j=j-1\;if\;i,j\neq 0\\ 0\;therwise \end{array}\right.$$ (1)

**Fig 4:**
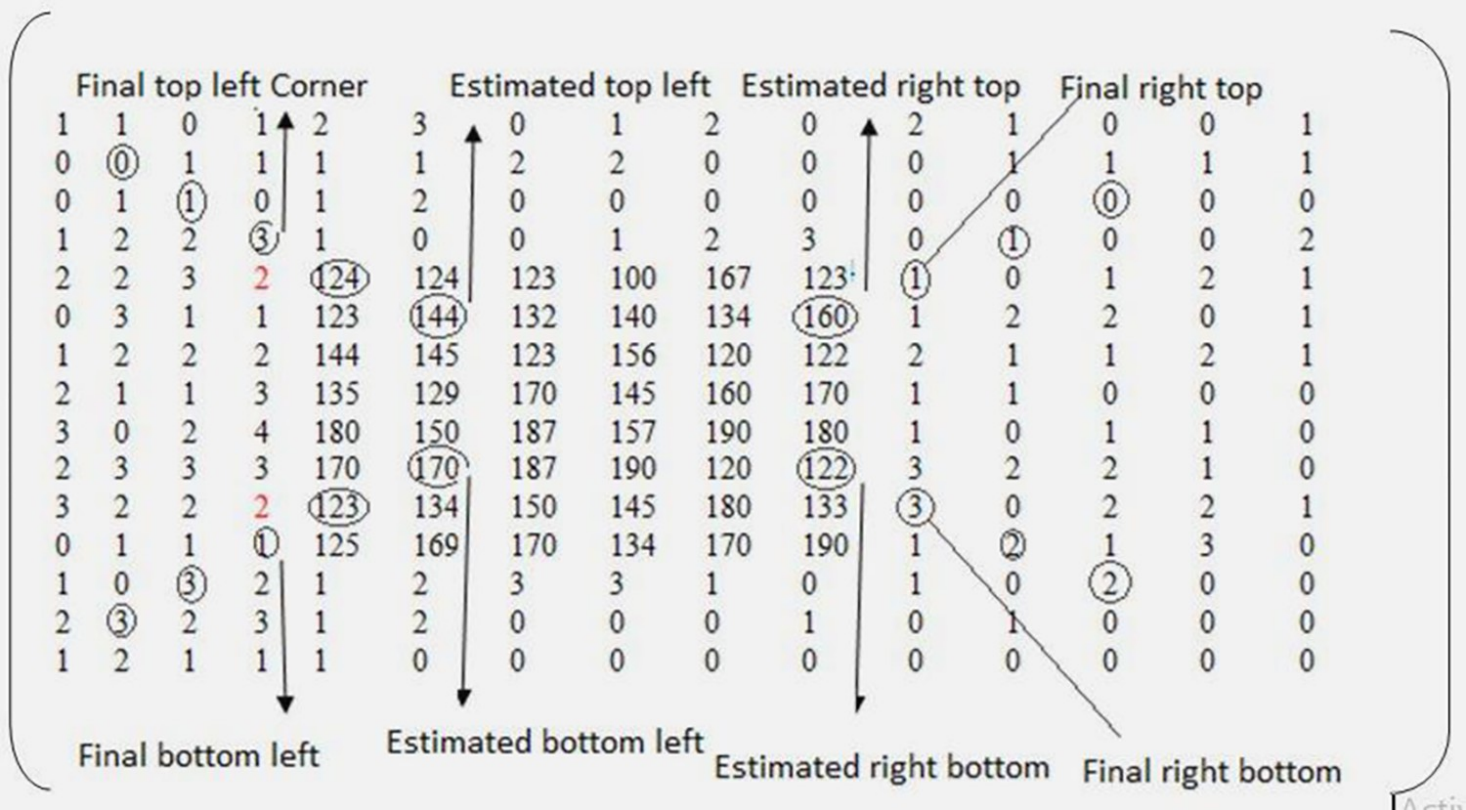
Extreme end point calculation for inner rectangle.

Where (*i*, *j*) refers to the location of pixel, and *I*(*i*, *j*) denotes to its grey level. To find the value of coordinate of the left-bottom end point, a negative sign is placed before the estimated border’s first value, i.e., [–20, 20]. This negative sign indicates to start row counting in upward direction starting from the last row till its well-defined bound. After this, it takes the estimated left-bottom point and starts counting for the final left-bottom point in the left downward side by matching the threshold with the estimated left-bottom point and its two diagonal pixels in the same way as described in Eq (2). To determine the coordinate value of the right-top endpoint, a negative sign is placed before the estimated border’s second value [20, –20]. The negative sign signifies to start counting from the last column to locate the right top’s initial value. Next, matching in right upward direction is initiated using Eq (2) and decision about the existence of top right corner border in the given image is taken. Next, negative sign is placed before the estimated corner border’s coordinates i.e., [–20, –20]. These negative signs indicate the direction, i.e., the initial coordinates of the right-bottom endpoint is generated by considering the last column as first column and last row as first row. Next, the final bottom coordinate of the inner rectangle is determined by performing comparison in the right downward direction using Eq (2). Once the coordinates of the four corner borders of the inner rectangle are computed then an extreme inner contour is drawn as shown in Fig 3(C). The area inside the extreme outer contour and extreme inner contour is then detached. The result of this module is shown in Fig 3(D). The working of this module is practically illustrated in Fig 4. We consider coordinate (5,5) as the maximum estimated top left endpoint for the extreme inner contour as shown in Fig 4. The procedure takes the pixel value 144 located at (5,5) and its previous two pixels values 124, and 3 located at (4,4) and (3,3), respectively, and match them with threshold value, i.e., T = 4. Its mathematical representation is shown as follows.

(144<4) and (124<4) and (3<4)

As this return false, so new point needs to be set for the left top corner, i.e., (4,4) and again the condition is checked. The procedure of comparison is continued until the given condition become true. The similar procedure is adopted to determine the coordinates of the other corner borders.

### B. Hairs removal

**Fig 5:**
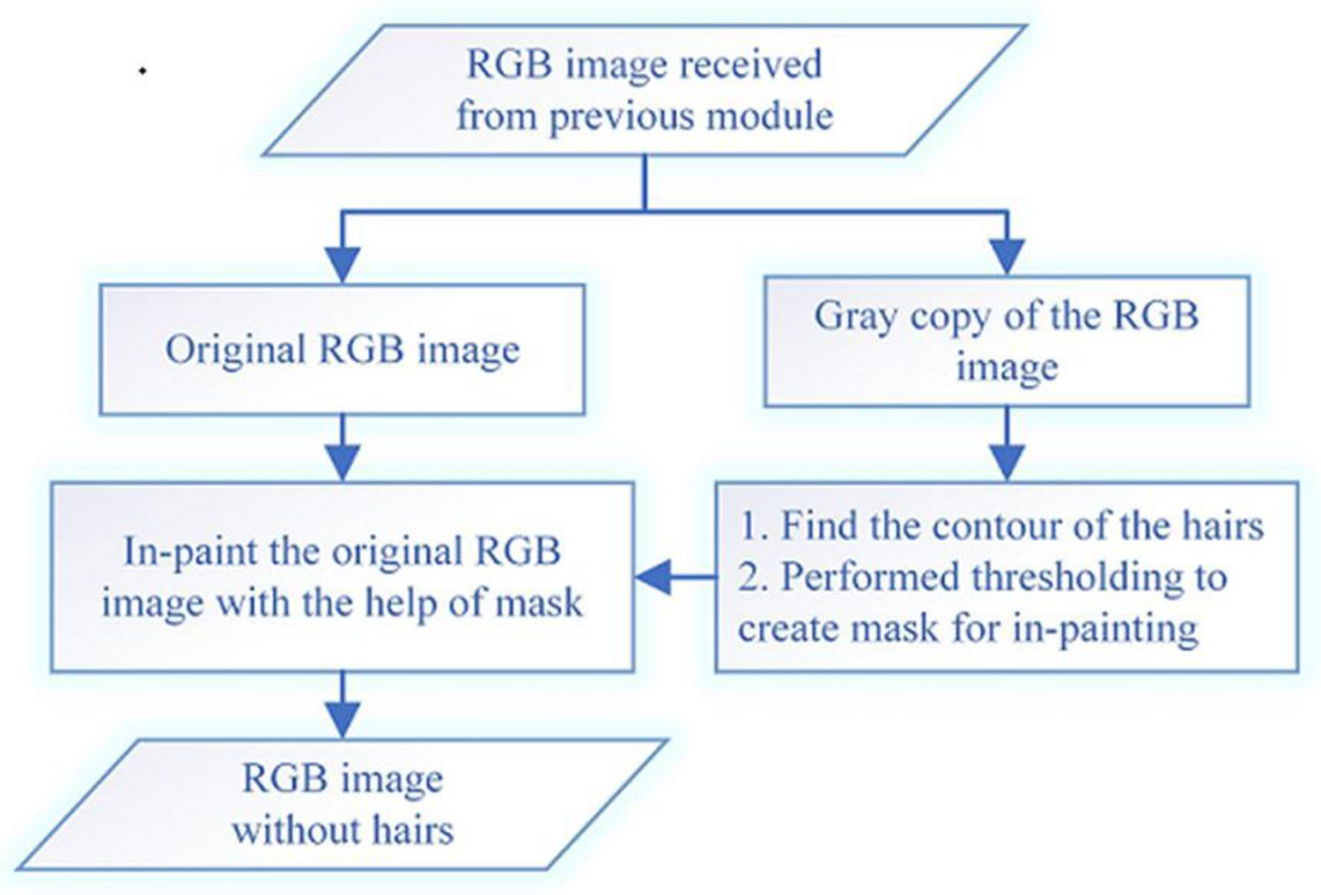
Workflow diagram of hairs removal process.

The skin hairs over the infected region in the dermoscopic images affect the lesion segmentation results. The goal of this module is to eliminate hairs from the resultant image of the prior module. The three steps used for the removal of hair are: hairs contour finding, mask creation, and removal of hairs from the image using in-painting. The work flow of hairs removal process is shown in the Fig 5. In the first step, a grey scale image of the RGB image obtained from the prior module is created. Next, the contours of the hairs on the grey scale image are detected by taking the difference of closing of the image and the image itself using Eq (2).


T(I)=(I●K)−I (2)

Where I is the grayscale image, K is the structural element, ● is the closing operation, and T(I) denotes to contour image. In the second step, a mask or threshold image is created from the contour image. For this purpose, a piecewise linear transformation function named as gray level slicing is applied on the contour image to identify the area to be in-painted in the image. The gray level slicing forms a mask of the contour image by transforming its pixels with values less than 10 to 0 and all of its other pixel values to 255. If the mask contains only the 0s, it means that image does not contain any hairs and thus this module will not carry out further computations that are required for hairs removal. On the other hand if the mask contains non–zero pixels then it will proceed towards third step because the non-zero pixels represent the area that is to be in-painted. In the third step, the previously created mask is used for in-painting to remove the hair from RGB image.

**Fig 6:**
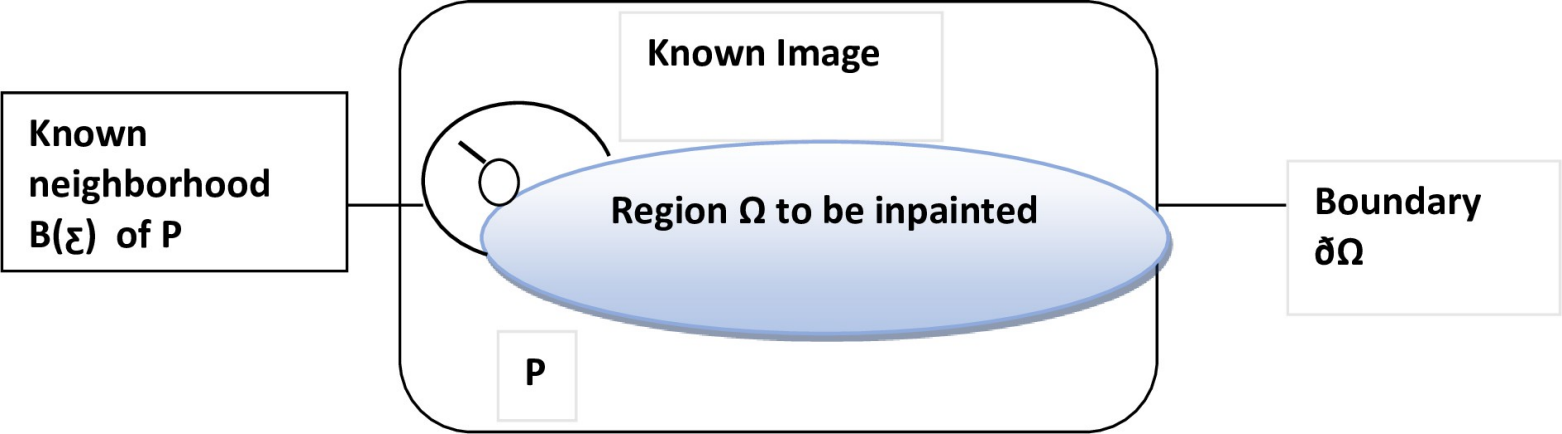
In-painting process of a point P.

The Fast-Marching method [28] is used for in-painting. To in-paint a point P located on the boundary (∂Ω) of the region (Ω) to be in-painted as shown in Fig 6. A neighbourhood B, of size ƹ (with value 2) of the pixels nearby P is taken. The first-order approximation I_q_(p) of the image at any point P, for small value of ƹ, image gradient ΔI(q) and I_q_ value at point P can be computed by Eq (3).


$$I_{q}(p)=I_{q}+\Delta I(q)(p-q)$$ (3)

Where I_q_, and ΔI(q) represent to any neighbour point of p, and gradient computed value by central difference, respectively. The point p is then in-paint as a function of all points q in Bƹ(p) by adding the estimates of all points q, weighted by a normalized weighting function w (q, p) using Eq (4)

$$I(p)=\frac{\sum_{q}\in B_{ƹ}(p)w(p,q)[I(q)+\Delta I(q)(p-q)]}{\sum_{q}\in B_{ƹ}(p)w(p,q)}$$ (4)

The w(p,q) weighting function is designed in such a way that the in-painting of point p propagates the value as well as sharp details of the image over Bƹ(p).

The weighting function is defined by the product of three terms using Eq (5)

w(p,q)=dir(p,q).dst(p,q).lev(p,q) (5)

The dir(p,q) denotes the directional components and ensure that the pixels participation nearby the normal direction N = ΔT, is more than those that are away from N. The dir(p, q) is defined by the Eq (6).


$$dir(p,q)=\frac{p-q}{\left|p-q\right|}.N(.p)$$ (6)

Where T denotes the distance Map, and N refers to normal to δΩ. The dst(p,q) in w(p,q) function refers to the geometric distance. The dst(p,q) is defined by Eq (7).


$$dst(p,q)=\frac{{d_{0}}^{2}}{{\left|p-q\right|}^{2}}$$ (7)

In Eq (7), the value of d_0_ is 1. The lev(p, q) of w(p,q) function denotes the level set distance component and it makes certain that pixels nearby contour of p are more contributed compared to those pixels which are away from p,. The value of this term can be computed using Eq (8).


$$lev(p,q)=\frac{T_{0}}{1+|T(p)-T(q)}$$ (8)

Where T_0 = 1_

**Fig 7:**
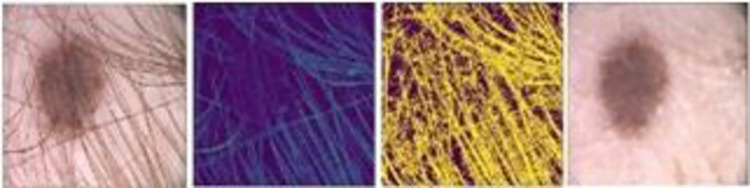
Hair removal process a) result of the previous module b) the contour of hair in Gray Scale Image c) Mask Computed from contour image d) RGB image after Hair removal.

The regions in the RGB image that are to be in-painted are identified with the help of mask. This module then starts from the boundary of the identified region and in-paint everything in boundary first and then in-paint the area inside the region. To do this, it gets a small neighbourhood of a pixel located at the boundary of a region and finds their normalized weighted sum. Weights selection is an important matter. The pixels placed near to the normal boundary, near to the point, and those placed over the boundary contours are given more weightage. The pixel intensity is replaced with the computed normalized weighted sum. This process is continued for each pixel in the region till all its pixels are in-painted. The same process is repeated for all regions in the whole image. The output of this module is shown in Fig 7.

### C. Image enhancement

This module aims to enhance the contrast of the resultant RGB image obtained from the previous module. As HSV color is more closely align with the way human visual system perceives color-making attributes [24], therefore, the RGB image is transformed into HSV color space using technique [29] first. In HSV image, the colors are represented by H and S, whereas the brightness of the pixels is reflected by V. It is observed that increase in brightness (values of V component) improves the image quality. So, this module enhances the V component, and H, S components remain unchanged.

**Fig 8:**
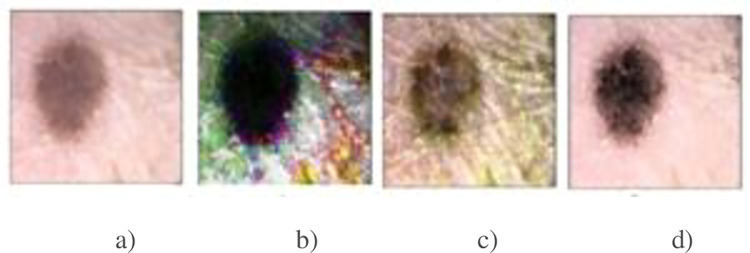
a) Original Image, b) Histogram equalization c) CLAHE d)modified histogram and log exponential transformation [16].

Different image enhancement approaches like Contrast Limited Adaptive Histogram Equalization(CLAHE), and histogram equalization [30] have been applied on the V component of the resultant RGB image. However, their results are not much effective as shown in Fig 8(B) and 8(C). In order to get better result, the modified histogram and log-exp transformation method proposed in [25] is applied on the V component of the resultant RGB image. This method of enhancement improves the low intensity of the image and avoids the significant decrement of the high intensity; thus, the contrast of the image is dynamically extended.

First, a function is developed to modify the histogram which smooths the color distribution without losing information. Second, a log-exp transformation is employed to increase the weighted values in the dark regions of the image and decrease those present in the bright regions. Third, a wider range for the brighter component is obtained with the application of nonlinear normalization. The enhanced version of the V-component is then combined with the H and S components to get a high contrast image as shown in Fig 8(D). For more details studies refer to [22].

### D. Image segmentation using GrabCut algorithm

The aim of this module to extract infected area in the resultant image of the prior module. The thresholding methods proposed in [31, 32] work fine for grey scale images, but their results on RGB images are poor as compared to GrabCut method [25]. The GrabCut method draws a rectangle to enclose the infected area in the image. It has been observed during the segmentation process that rectangle size 12% less from the original image with the ratio of 5% from the top left or starting point and 7% from the right bottom or end point is more effective for segmenting the infected region. The Eqs (9) and (10) are used to compute the size of rectangle.


$$A=int\left(\frac{image.shape[1]\times 5}{100}\right)$$ (9)

$$B=int\left(\frac{Image.shape[0]\times 5}{100}\right)$$ (10)

Where A, and B are the starting point of the rectangle. For computing the end points replace 5 with 7 and subtract the result from height and width of the image. For instance, image with size 430 × 430, the size of rectangle is (21, 21,400, 400) used to segment the lesion from the normal skin. Next, a tri-map is initialized to perform the initial labelling. The labelling performed using tri-map is defined by Eq (11).


$$T=\{T_{B},T_{u},T_{F}\}$$ (11)

**Fig 9:**
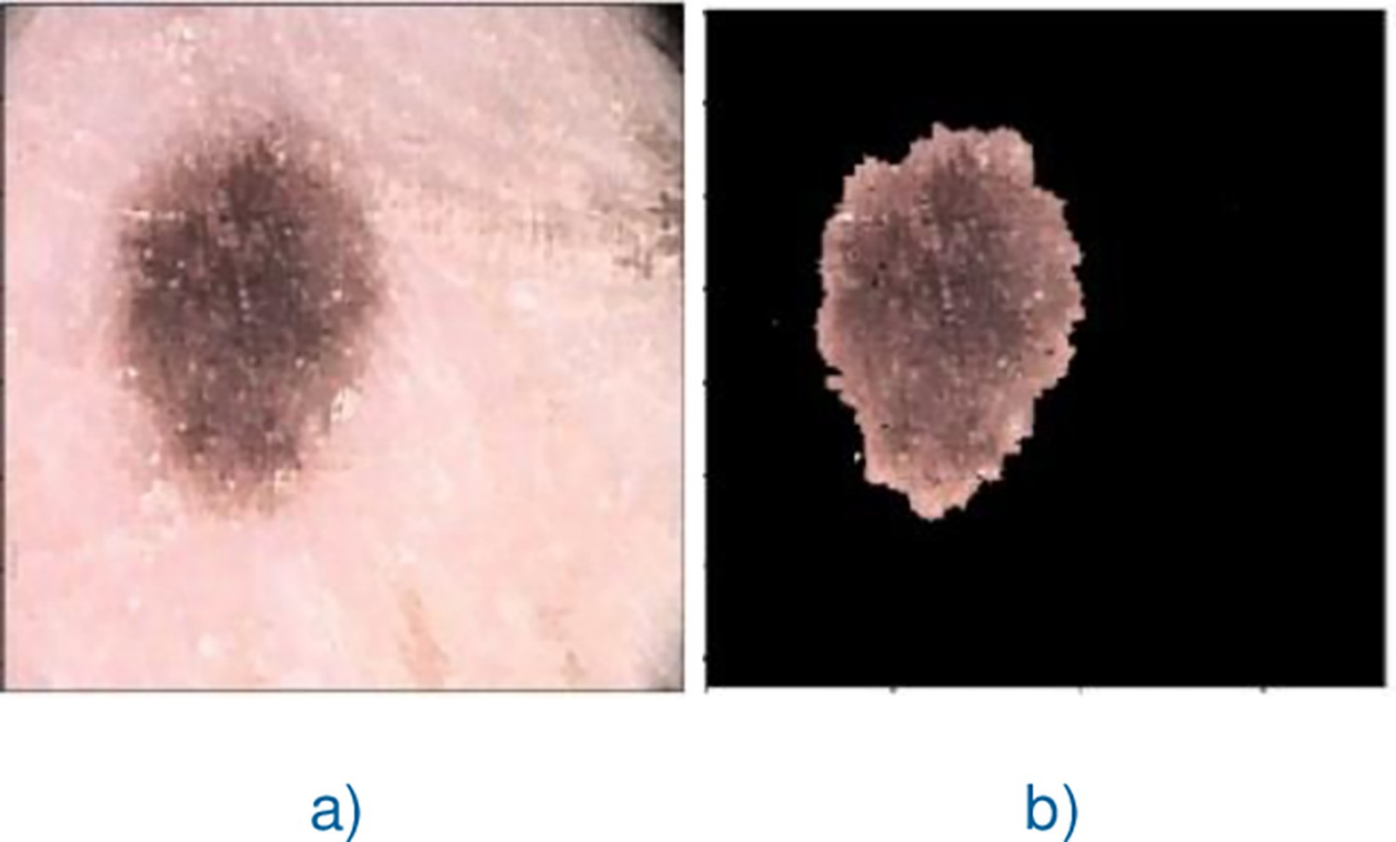
a) Input obtained from previous module b) Results of segmentation module.

Where T_B,_ T_u,_ and T_F_ are the variables which store the background, the unknown and the foreground pixels, respectively. The value of T_u_ is computed by complementing the T_B_, i.e., $T_{u}=\bar{T_{B}}$ and T_F_ = Φ. After this, two Gaussian Mixture Models (GMM) are generated. The First one is created for T_B_ with an = 0, and the second one is created for T_u_ with an = 1. The GrabCut then assigns new labels by computing new GMMs both for background and foreground and removes the old ones. A new pixel distribution is initiated, and graph is constructed from it with pixels represented by graph nodes. Every pixel connects to any two leaf nodes defined as sink or background node and source or foreground node. After graph weights initialization, min-cut is applied on the graph to compute new values for the pixels on Tu. The value shift from 1 to 0 or 0 to 1 in each iteration. This process is continued until the convergence of the labelling of the foreground and background pixels. The result of segmentation is shown in Fig 9.

## 4. Results and discussion

The dataset’s details, information regarding evaluation metrics that are used for performance checking, and the results’ comparison are provided in section.

### A. Dataset

In this research work, two datasets, i.e., Hospital Pedro Hispano (PH2) [33] and ISIC2018 [34] were used for the results evaluation of our proposed method. The PH2 dataset contains 435 dermoscopy skin images, and their ground truths. The dimension of dataset’s images is 572 × 765, and their file format is BMP. The ISIC2018 dataset contains 2500 images in JPG format with ground truth. We have collected 200 images randomly from each of the aforementioned datasets to test the results’ accuracy of the proposed method. Our proposed method was implemented in Python 3.0 running on Intel(R) Core(TM) i5-4300U CPU @ 1.90GHz 2.49 GHz computer with 4.00 GB RAM, and 500GB hard disk.

### B. Evaluation metrics

The Jaccard index and dice index were used as evaluation metrics for checking the proposed method’s performance. These two metrics were chosen because most of the researchers used them for evaluation purpose in the context of skin lesion segmentation. The Jaccard index is used to measures the overlap between two images using Eq (12). Like Jaccard index, the dice index computes the similarity index between the given images using Eq (13).


$$Jaccard\;Index=\frac{TP}{TP+FP+FN}$$ (12)

$$Dice\;Index=\frac{2TP}{\left(FP+TP)+(TP+FN\right)}$$ (13)

Where TP, and FP refers to lesion pixels extracted as lesion pixels, and non-lesion pixels extracted as lesion pixel, respectively, while FN, and TN represent lesion pixels extracted as non-lesion pixels, and non-lesion pixels extracted as non-lesion pixels, respectively.

### C. Comparison

In this subsection, the performance comparison of our proposed method with state-of-art segmentation methods is presented. The performance of our proposed method and those present in the literature were tested on 200 images taken from each of the aforementioned datasets. The accuracy of the results of lesion segmentation from skin dermoscopic images mainly depends on three factors, i.e., border removal, hair removal, and enhancement technique applied. The application status of these three factors by available and our proposed methods are listed in Table 1.

**Table 1:** Application status of border removal, hair removal, and enhancement technique.

Methods	Border Removal	Hair Removal	Enhancement
Adaptive thresholding [8]	No	No	Yes
Hybrid thresholding [9]	Yes	Yes	No
Ensembles of thresholding [11]	No	No	Yes
Grabcut [5]	No	No	Yes
Deep residual network [24]	No	No	No
Delaunay Triangulation [13]	No	Yes	Yes
Perceptual Color Difference Saliency [14]	Yes	Yes	Yes
Cohen–Daubechies–Feauveau biorthogonal wavelet [4]	Yes	Yes	No
Adaptive automatic thresholding [10]	No	No	No
novel hybrid approach [23]	No	Yes	No
optimized fire fly algorithm [19]	No	Yes	No
**Our proposed method**	**Yes**	**Yes**	**Yes**

As methods [5, 8, 11–14, 19, 23] do not deal with images containing artifacts like hairs, and borders and the PH2 dataset contains images with borders and hairs, therefore their results’ accuracy in term of Jaccard index and dice index are not satisfactory.

The results’ accuracy of [4, 9] is better than [5, 8, 11–14, 19, 23] because they employ artifact removal techniques. However, their accuracy is smaller than our proposed method because they blur the image after hair removal which affects the results of lesion segmentation. Furthermore, their border removal methods are not much effective in case of images containing varying size borders.

The method in [14] uses a border removal method, but its results’ accuracy is unsatisfactory for images with varying size corner borders. Additionally, it blurs the image after hair removal.

Our proposed method of lesion segmentation achieved comparatively better results in terms of Jaccard index, and dice index as shown in Table 2. This is because of the reason that our proposed method uses border removal method capable of removing not only the varying size corner borders but also the corner borders with color similar to lesion. Furthermore, the skin hair removal method of our proposed method creates comparatively less blurring during the hair removal process. Next, our proposed method employs state-of-the-art enhancement method to highlight the lesion from the normal skin.

**Table 2:** Quantitative lesion segmentation results.

Method	PH2		ISIC 2018	
	Jac-card Index	Dice Index	Jac-card Index	Dice Index
Adaptive thresholding and Skewness correction [8]	0.68	0.70	0.70	0.73
Border detection in dermoscopy images using hybrid thresholding [9]	0.64	0.69	0.69	0.71
Ensembles of thresholding [11]	0.67	0.70	0.68	0.72
Grabcut [5]	0.68	0.70	0.78	0.80
Deep residual network [24]	0.54	0.58	0.56	0.61
Delaunay Triangulation [13]	0.70	0.71	0.71	0.70
Perceptual Color Difference Saliency [14]	0.66	0.71	0.69	0.72
Cohen–Daubechies–Feauveau biorthogonal wavelet [4]	0.73	0.75	0.74	0.73
Adaptive automatic thresholding [10]	0.63	0.68	0.65	0.69
novel hybrid approach [23]	0.67	0.65	0.70	0.69
optimized fire fly algorithm [19]	0.68	0.69	0.71	0.70
**Our proposed method**	**0.77**	**0.87**	**0.80**	**0.82**

**Fig 10:**
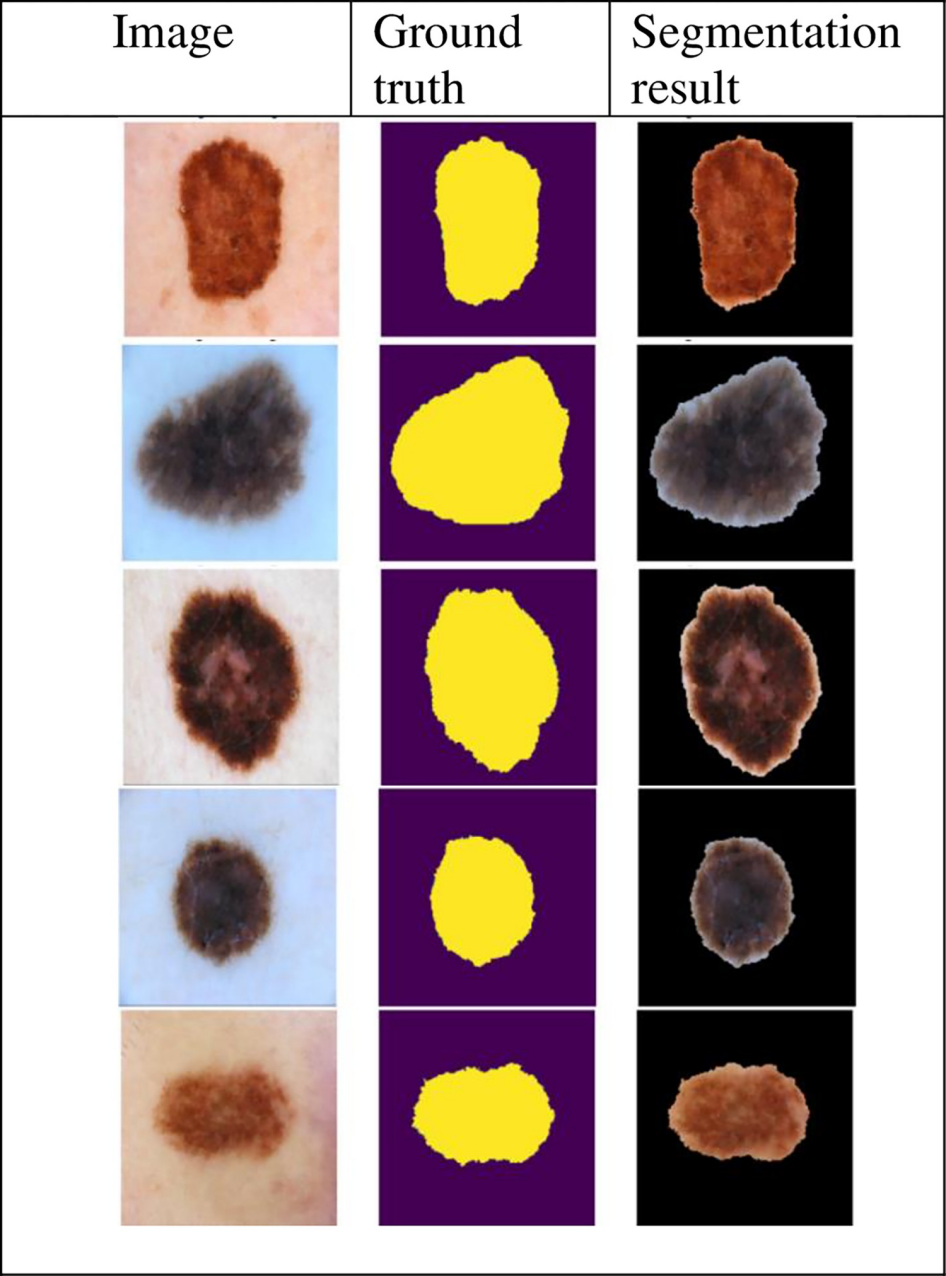
Sample results of our proposed method.

The ISIC 2018 is preprocessed dataset containing images with no corner borders. The segmentation accuracy of our proposed segmentation method on this dataset is better than [5, 8, 11–14, 19, 23], because of employing state-of-the-art image enhancement and segmentation technique, while its segmentation accuracy is comparable to [4, 9, 14] because of using powerful image enhancement technique and better corner border and hair removal methods. Some of the results of our proposed method on images taken from ISIC 2018 dataset are shown in Fig 10.

**Fig 11:**
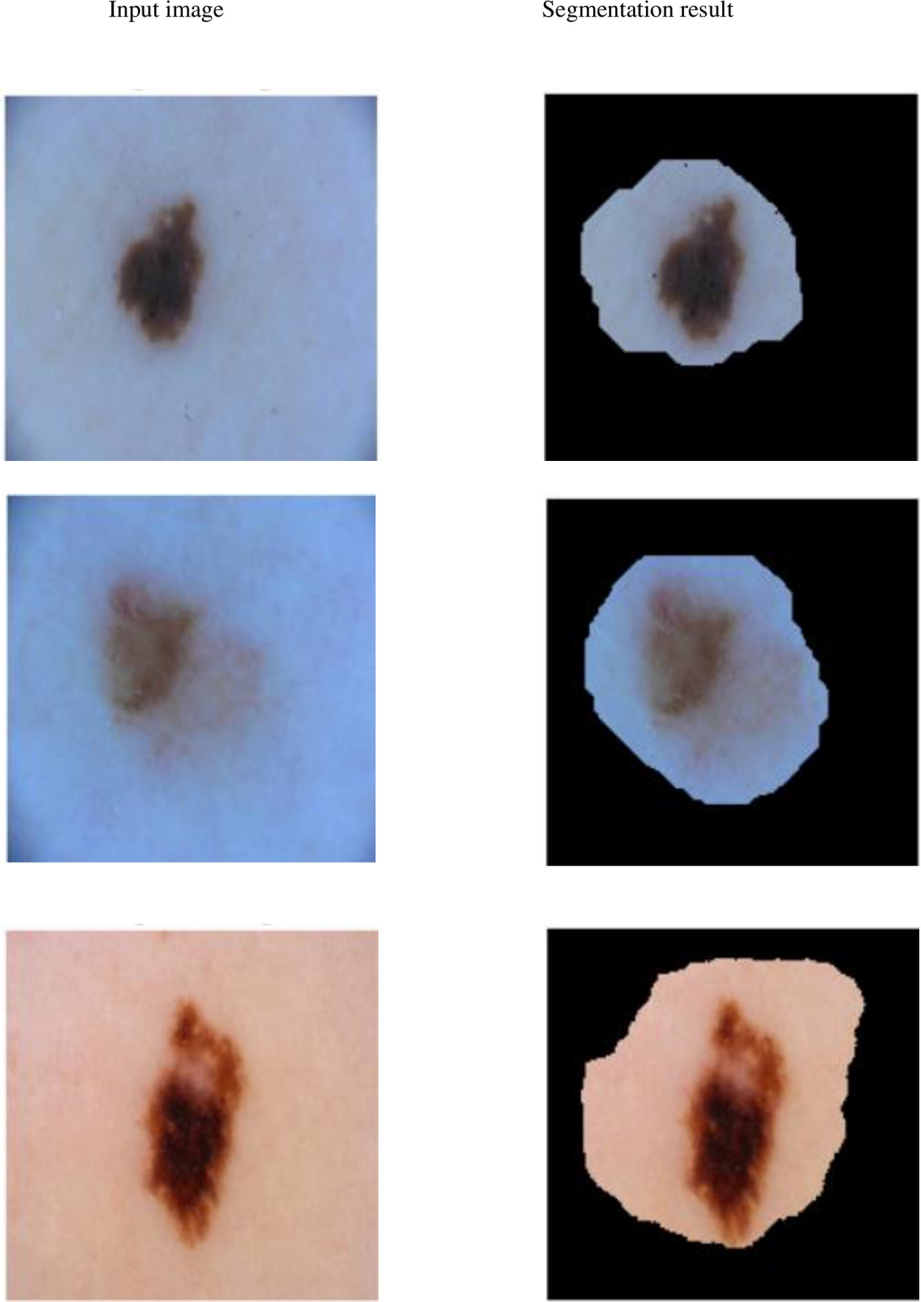
Results of our proposed method over dermoscopic images taken from ISIC 2018 dataset containing small size infected region.

The results of our proposed segmentation method are not satisfactory for images containing small infected region as shown in Fig 11. This is because of the reason that our proposed method forms an automatic rectangle needed for GrabCut method. The GrabCut method includes part of background area of the dermoscopic image with the resultant infected area due to inappropriate size of rectangle.

## 5. Conclusions

The available skin lesion segmentation approaches often generate false lesion segmentation results for dermoscopic images containing varying size corner borders, or color borders with color similar to lesion. To cope with this issue, our proposed machine learning based skin lesion segmentation method utilizes a novel border removal method capable of removing varying size corner borders, and/or color borders with color similar to lesion and thus improve lesion segmentation results.

Also, it caters to the problem of annoying artifacts, like hairs using a comparatively better hair removal method. The contrast between the lesion and its surrounding is then improved by employing state-of-the-art image enhancement method which contributes towards accurate lesion segmentation. Our proposed lesion segmentation method achieved comparatively better Jaccard Index, and Dice index values on PH2, and ISIC2018 datasets containing images with varying size corner borders with color similar to lesion and/or hair due to the application of novel borders and hair removal methods. The GrabCut method causes over-segmentation in case of the dermoscopic images containing small size lesion due to the selection of inappropriate rectangle size used for initialization of Grabcut method. In future, we intend to device a method for appropriate selection of rectangle to avoid over-segmentation in case of dermoscopic images containing small size lesion.

## References

[1] Ge Z., Demyanov S., Chakravorty R., Bowling A., and Garnavi R., “Skin Disease Recognition Using Deep Saliency Features and Multimodal Learning of Dermoscopy and Clinical Images,” 2017, pp. 250–258. doi: 10.1007/978-3-319-66179-7_29

[2] “Melanoma skin cancer report. The Global Coalition for Melanoma Patient Advocacy,” *https://melanomapatients.org.au/wp-content/uploads/2020/04/2020-campaign-report-GC-version-MPA_1.pdf*., 2020.

[3] Rogers H. W., Weinstock M. A., Feldman S. R., and Coldiron B. M., “Incidence Estimate of Nonmelanoma Skin Cancer (Keratinocyte Carcinomas) in the US Population, 2012,” *JAMA Dermatology*, vol. 151, no. 10, p. 1081, Oct. 2015, doi: 10.1001/jamadermatol.2015.1187 25928283

[4] Khalid S. et al., “Segmentation of skin lesion using Cohen–Daubechies–Feauveau biorthogonal wavelet,” *Springerplus*, vol. 5, no. 1, p. 1603, Dec. 2016, doi: 10.1186/s40064-016-3211-4 27652176 PMC5028360

[5] Fakrul I. T., “Automatic Skin Lesion Segmentation Using GrabCut in HSV Colour Space.,” *Computer Vision and Pattern Recognition*, 2018.

[6] Mocellin S. and Nitti D., “Cutaneous Melanoma In Situ: Translational Evidence from a Large Population-Based Study,” *The Oncologist*, vol. 16, no. 6, pp. 896–903, Jun. 2011, doi: 10.1634/theoncologist.2010-0340 21632457 PMC3228223

[7] Lucas C. R., Sanders L. L., Murray J. C., Myers S. A., Hall R. P., and Grichnik J. M., “Early melanoma detection: Nonuniform dermoscopic features and growth,” *J Am Acad Dermatol*, vol. 48, no. 5, pp. 663–671, May 2003, doi: 10.1067/mjd.2003.283 12734494

[8] Sforza G. et al., “Using Adaptive Thresholding and Skewness Correction to Detect Gray Areas in Melanoma In Situ Images,” *IEEE Transactions on Instrumentation and Measurement*, vol. 61, no. 7, pp. 1839–1847, Jul. 2012, doi: 10.1109/TIM.2012.2192349

[9] Garnavi R., Aldeen M., Celebi M. E., Varigos G., and Finch S., “Border detection in dermoscopy images using hybrid thresholding on optimized color channels,” *Computerized Medical Imaging and Graphics*, vol. 35, no. 2, pp. 105–115, Mar. 2011, doi: 10.1016/j.compmedimag.2010.08.001 20832992

[10] G., M. A. M.–E. C. G. V. and Rahil S. F., “A Robustness Segmentation Approach for Skin Cancer Image Detection Based on an Adaptive Automatic Thresholding Technique,” *American Journal of Intelligent Systems*, vol. 35, no. 2, pp. 105–115, 2011.

[11] -M. C., Q. W. S. H. H. I. and Emre G. S., “Lesion Border Detection in Dermoscopy Images Using Ensembles of Thresholding Methods,” *Skin Research & Technology*, vol. 19, no. 1, pp. 252–58, 2012. 10.1111/j.1600-0846.2012.00636.x 22676490

[12] Y., H. C. Q. D. J. Q. and Lequan P. H., “Automated melanoma recognition in dermoscopyimages via very deep residual networks,” *IEEE TRANSACTIONS ON MEDICAL IMAGING*, vol. 36, no. 4, pp. 994–04, 2017. doi: 10.1109/TMI.2016.2642839 28026754

[13] Pennisi A., Bloisi D. D., Nardi D., Giampetruzzi A. R., Mondino C., and Facchiano A., “Skin lesion image segmentation using Delaunay Triangulation for melanoma detection,” *Computerized Medical Imaging and Graphics*, vol. 52, pp. 89–103, Sep. 2016, doi: 10.1016/j.compmedimag.2016.05.002 27215953

[14] Olugbara O. O., Taiwo T. B., and Heukelman D., “Segmentation of Melanoma Skin Lesion Using Perceptual Color Difference Saliency with Morphological Analysis,” *Mathematical Problems in Engineering*, vol. 2018, pp. 1–19, 2018, doi: 10.1155/2018/1524286

[15] R D S. and A S., “Deep Learning Based Skin Lesion Segmentation and Classification of Melanoma Using Support Vector Machine (SVM),” *Asian Pacific Journal of Cancer Prevention*, vol. 20, no. 5, pp. 1555–1561, May 2019, doi: 10.31557/APJCP.2019.20.5.1555 31128062 PMC6857898

[16] Liu L., Tsui Y. Y., and Mandal M., “Skin Lesion Segmentation Using Deep Learning with Auxiliary Task,” *Journal of Imaging*, vol. 7, no. 4, p. 67, Apr. 2021, doi: 10.3390/jimaging7040067 34460517 PMC8321325

[17] -S. Y., B.–A. A. N.-B. P. G.-R. H. A. M. and · Mohamed K. S., “Deep learning based an automated skin lesion segmentation and intelligent classification model,” *Journal of Ambient Intelligence and Humanized Computin*, vol. 12, pp. 3245–55, 2020.

[18] –F. M., T. M. and Pedro P. R., “A widespread of algorithms for automatic segmentation of dermoscopic images,” *Lecture Notes in Computer Science*, vol. 7887, pp. 592–99, 2013.

[19] Garg S. and Jindal B., “Skin lesion segmentation using k-mean and optimized fire fly algorithm,” *Multimedia Tools and Applications*, vol. 80, no. 5, pp. 7397–7410, Feb. 2021, doi: 10.1007/s11042-020-10064-8

[20] Sarker Md. M. K. et al., “SLSDeep: Skin Lesion Segmentation Based on Dilated Residual and Pyramid Pooling Networks,” 2018, pp. 21–29. doi: 10.1007/978-3-030-00934-2_3

[21] Bi L., Kim J., Ahn E., Kumar A., Fulham M., and Feng D., “Dermoscopic Image Segmentation via Multistage Fully Convolutional Networks,” *IEEE Transactions on Biomedical Engineering*, vol. 64, no. 9, pp. 2065–2074, Sep. 2017, doi: 10.1109/TBME.2017.2712771 28600236

[22] Lin B. S., Michael K., Kalra S., and Tizhoosh H. R., “Skin lesion segmentation: U-Nets versus clustering,” in *2017 IEEE Symposium Series on Computational Intelligence (SSCI)*, Nov. 2017, pp. 1–7. doi: 10.1109/SSCI.2017.8280804

[23] Singh L., Janghel R. R., and Sahu S. P., “SLICACO: An automated novel hybrid approach for dermatoscopic melanocytic skin lesion segmentation,” *International Journal of Imaging Systems and Technology*, vol. 31, no. 4, pp. 1817–1833, Dec. 2021, doi: 10.1002/ima.22591

[24] Yu L., Chen H., Dou Q., Qin J., and Heng P.-A., “Automated Melanoma Recognition in Dermoscopy Images via Very Deep Residual Networks,” *IEEE Transactions on Medical Imaging*, vol. 36, no. 4, pp. 994–1004, Apr. 2017, doi: 10.1109/TMI.2016.2642839 28026754

[25] Zhuang L. and Guan Y., “Image Enhancement Using Modified Histogram and Log-Exp Transformation,” *Symmetry (Basel)*, vol. 11, no. 8, p. 1062, Aug. 2019, doi: 10.3390/sym11081062

[26] Kim H.-J., Lee J.-M., Lee J.-A., Oh S.-G., and Kim W.-Y., “Contrast Enhancement Using Adaptively Modified Histogram Equalization,” 2006, pp. 1150–1158. doi: 10.1007/11949534_116

[27] Rother C., Kolmogorov V., and Blake A., “‘GrabCut: Interactive Foreground Extraction Using Iterated Graph Cuts’” *ACM Transactions on Graphics*, vol. 23, no. 3, pp. 309–314, Aug. 2004, doi: 10.1145/1015706.1015720

[28] Alexandru T., “An Image Inpainting Technique Based on the Fast Marching Method,” *Journal of Graphics Tools*, vol. 9, no. 1, pp. 23–24, 2004.

[29] Irvnriir, “Wikipedia.,” 2021. https://en.wikipedia.org/wiki/HSL_and_HSV. (accessed Feb. 03, 2022).

[30] A A H. T., R M., M M. S., and S A., “Comparison of Different Segmentation Algorithms for Dermoscopic Images,” *ICTACT Journal on Image and Video Processing*, vol. 5, no. 4, pp. 1030–1036, May 2015, doi: 10.21917/ijivp.2015.0151

[31] Kapur J. N., Sahoo P. K., and Wong A. K. C., “A new method for gray-level picture thresholding using the entropy of the histogram,” *Computer Vision*, *Graphics*, *and Image Processing*, vol. 29, no. 3, pp. 273–285, Mar. 1985, doi: 10.1016/0734-189X(85)90125-2

[32] Kittler J. and Illingworth J., “Minimum error thresholding,” *Pattern Recognition*, vol. 19, no. 1, pp. 41–47, Jan. 1986, doi: 10.1016/0031-3203(86)90030-0

[33] T. Mendonca, P. M. Ferreira, J. S. Marques, A. R. S. Marcal, and J. Rozeira, “A dermoscopic image database for research and benchmarking,” in *2013 35th Annual International Conference of the IEEE Engineering in Medicine and Biology Society (EMBC)*, Jul. 2013, pp. 5437–5440. doi: 10.1109/EMBC.2013.6610779 24110966

[34] Codella N. et al., “Skin Lesion Analysis Toward Melanoma Detection 2018: A Challenge Hosted by the International Skin Imaging Collaboration (ISIC),” Feb. 2019.

