# Correction: Machine learning based skin lesion segmentation method with novel borders and hair removal techniques

**DOI:** 10.1371/journal.pone.0316115

**Published:** 2024-12-17

**Authors:** Mohibur Rehman, Mushtaq Ali, Marwa Obayya, Junaid Asghar, Lal Hussain, Mohamed K. Nour, Noha Negm, Anwer Mustafa Hilal

There are errors in the Funding statement. The correct Funding statement is as follows: The authors extend their appreciation to the Deanship of Scientific Research at King Khalid University for funding this work through Larg Groups Project under grant number (RGP2/42/43). Princess Nourah bint Abdulrahman University Researchers Supporting Project number (PNURSP2022R203), Princess Nourah bint Abdulrahman University, Riyadh, Saudi Arabia. The authors would like to thank the ‎Deanship of Scientific Research at Umm Al-Qura University ‎for supporting this work by Grant Code: (22UQU4310373DSR39). The funders had no role in study design, data collection and analysis, decision to publish, or preparation of the manuscript.
